# The CDC Worksite Health ScoreCard: A Tool to Advance Workplace Health Promotion Programs and Practices

**DOI:** 10.5888/pcd19.210375

**Published:** 2022-06-23

**Authors:** Enid Chung Roemer, Karen B. Kent, Ron Z. Goetzel, John Krill, Farrah Spellman Williams, Jason E. Lang

**Affiliations:** 1 Institute for Health and Productivity Studies, Johns Hopkins Bloomberg School of Public Health, Baltimore, Maryland; 2 National Center for Chronic Disease Prevention and Health Promotion, Centers for Disease Control and Prevention, Atlanta, Georgia

## Abstract

**Introduction:**

The CDC Worksite Health ScoreCard (ScoreCard) is a free, publicly available survey tool designed to help employers assess the extent to which they have implemented evidence-based interventions or strategies at their worksites to improve the health and well-being of employees. We examined how, how broadly, and to what effect the ScoreCard has been applied.

**Methods:**

We analyzed peer-reviewed and grey literature along with the ScoreCard database of online submissions from January 2012 through January 2021. Our inclusion criteria were workplace settings, adult working populations, and explicit use of the ScoreCard.

**Results:**

We found that the ScoreCard had been used in 1) surveillance efforts by states, 2) health promotion training and technical assistance, 3) research on workplace health promotion program effectiveness, and 4) employer efforts to improve program design, implementation, and evaluation.

**Conclusion:**

The ScoreCard has been used as intended to support the development, planning, monitoring, and continuous improvement of workplace health promotion programs. Our review revealed gaps in the tool and opportunities to improve it by 1) enhancing surveillance efforts, 2) engaging employers in low-wage industries, 3) adding new questions or topic areas, and 4) conducting quantitative studies on the relationship between improvements in the ScoreCard and employee health and well-being outcomes.

**Summary**

**What is already known on this topic?**

The CDC ScoreCard is a tool designed to help employers evaluate the extent to which they have implemented evidence-based interventions to improve worker health, safety, and well-being.

**What is added by this report?**

Our study found that the ScoreCard has been used effectively as designed, but also revealed gaps and opportunities to improve its usefulness.

**What are the implications for public health practice?**

The ScoreCard can be leveraged as a surveillance tool to track employer efforts to improve population health alongside business initiatives, especially in low-wage industries. More research is needed to illustrate a causal link between improvements in organizational health, improvements in employee health, and business outcomes.

## Introduction

The US spends billions of dollars treating chronic diseases but directs few resources to proactively address the health, safety, and well-being of working adults (1). To improve population health, public health agencies should consider engaging adults where they spend most of their waking hours — at work. Modifiable health risk factors (eg, poor nutrition, physical inactivity, obesity, smoking) contribute to many diseases and disorders, including high blood pressure, high cholesterol, diabetes, heart disease, stroke, and various forms of cancer (2). Additionally, studies have shown that evidence-based workplace health promotion (hereinafter, workplace health) programs can reduce employee health risks, thereby improving performance and reducing health care costs (2,3).

The challenges of the COVID-19 pandemic heightened employers’ awareness of and need for effective workplace health programs. The pandemic revealed the impact of social determinants of health (SDOH) (ie, economic stability, access to and quality of education, health care access and quality, neighborhood and built environment, and social and community context), with disproportionately high rates of health risk factors and chronic illnesses among segments of the population that are disadvantaged in these areas, particularly racial and ethnic minority populations. For example, 9% of low-wage workers, who often work in service and health care industries that put them in close contact with coworkers and the public, reported being in fair or poor health, which in turn puts them at greater risk for becoming seriously ill if they contract COVID-19 (4). The pandemic also brought about an uptick in mental health problems (eg, anxiety, depression, suicidal thoughts, social isolation, substance use disorders) (5). Altogether, these challenges spotlighted the importance of public health preparedness and emergency response in maintaining business continuity supported by a healthy and productive workforce.

Employers seek assistance from the public health community to guide them in building and sustaining healthy workforces and communities. In response, the Centers for Disease Control and Prevention (CDC) developed the CDC Worksite Health ScoreCard (hereinafter, ScoreCard) (6) to guide employers in improving employee health, safety, and well-being by adopting evidence-based workplace health programs. The ScoreCard currently covers a broad range of evidence-based strategies across 18 topic areas (eg, organizational supports, physical activity, nutrition, prediabetes and diabetes, stress management, alcohol and other substance use), which employers can use to identify program gaps and set priorities. Overall, the ScoreCard has 154 yes/no questions. Each question represents an individual intervention, strategy, or action an employer can take to improve employee health, such as lifestyle counseling services, physical and social environmental supports, workplace policies, and design of health plan benefits.

The ScoreCard is available as an online questionnaire on the CDC website (www.cdc.gov) and as a downloadable file (PDF) for users to complete by hand and self-score (6). The online platform requires users to register with 2 types of accounts (administrator, worksite) so that employers with multiple business units can complete the survey at the local level, given that program offerings may differ by location. On submitting a completed survey, employers receive automatic access to their scores and a series of benchmarking reports in their account dashboard. The online system records all submitted ScoreCards so that employers have a history of their organizational capacity for implementing workplace health initiatives and can monitor progress over time. Additionally, the CDC website provides many tools and resources, such as user guides, video tutorials for interpreting results, and the Action Planning Tool, a 3-step process to help employers identify and prioritize strategies and next steps to improve their workplace health program.

The ScoreCard, which consists of 12 topic areas, was introduced in 2012. Since then, it has been updated twice: in 2014 (4 new topics added) and 2019 (2 new topics added). The updates were made to ensure that the tool remained current, evidence-based, and valid. Psychometric analyses have shown it to be a reliable and valid instrument to assess organizational health at the worksite (7). However, the tool has not been fully studied in terms of its effectiveness in improving workplace health offerings and, in turn, worker health, safety, and well-being. To fill that gap, through an examination of peer-reviewed studies, grey literature, and a database of ScoreCard users (summary data provided by CDC via email communications based on their online survey submissions, January 25, 2021), we report on 1) how the ScoreCard is currently used by employers across the US, 2) the current state of scientific evidence and practical applications for the instrument, 3) gaps in knowledge, and 4) opportunities for improving future design and utility, especially as the nature of work is rapidly evolving, largely because of the pandemic.

## Methods

### Scientific literature

Our literature review consisted of published studies that referred to the ScoreCard in their methods or outcomes (Table 1). We included peer-reviewed articles that described studies of adults aged 18 to 64 years in a workplace setting. Articles could be case studies or state reports.

**Table 1:** Literature Review Criteria, CDC Worksite Health ScoreCard

Criteria	Included	Excluded
Publication type/status	Peer-reviewed journal articles (published or accepted) Case studies State reports	Conference abstracts Nonacademic websites
Population/setting	Adults (18–64 y) Workplace	Children Seniors
Interventions	Explicit use of the CDC Worksite Health ScoreCard (6)	No explicit use of the CDC Worksite Health ScoreCard, even if similar organizational health assessments were used
Outcomes	Health behaviors of employees Health risk profile of employees Workplace health program offerings/participation CDC Worksite Health ScoreCard score	NA
Publication date	January 2012 to January 2021	Before January 2012 or after January 2021

Abbreviation: NA, not applicable; CDC, Centers for Disease Control and Prevention.

Applying these inclusion/exclusion criteria, we conducted a literature search of academic databases, including PubMed, EBSCO Academic Search Complete, PsycINFO, and Google Scholar, for articles published from January 2012 through January 2021. In these searches, we used the following keywords: “CDC Worksite Health ScoreCard,” “CDC ScoreCard,” and “CDC Worksite.” This search yielded 12 relevant peer-reviewed articles (Table 2).

**Table 2:** Summary of Peer-Reviewed Literature, CDC Worksite Health ScoreCard

Author, year (reference)	Worksites, populations, and interventions studied	Demonstrated ScoreCard effectiveness, outcomes, and implications
Cluff et al, 2018 (8)	Reports on the CDC’s Work@Health Program, which used the CDC Worksite Health ScoreCard (ScoreCard) (6,9). An 8-module training curriculum was used to guide program managers on the essential elements of evidence-based workplace health promotion programs with the ScoreCard as a guide for effective programming.	Demonstrated that ScoreCard use combined with training and technical support can improve employers’ knowledge about workplace health promotion and significantly increase the number of evidence-based health interventions in place at their worksites.
Gutermuth et al, 2018 (10)	Identified 18 worksites with published studies related to their health promotion programs. Used the ScoreCard as a framework to summarize information on the organizational supports and physical activity strategies that these worksites had in place.	Of the 18 worksite health promotion programs examined, 11 produced significant improvements in physical activity. Incentives, health risk assessments, health promotion committees, leadership support, marketing, and subsidies or discounts for the use of exercise facilities were the most effective organizational supports cited, and physical activity seminars, classes, and workshops were the most effective physical activity strategies cited. The ScoreCard provided a practical framework for evaluating programs and interpreting the findings.
Henke et al, 2019 (11)	Examined the relationship between internal and external cultures of health scores and changes to employees’ health risks, health care use, and costs for 21 large employers (N = 641,901 employees).	Improvements in the internal culture of health (based on ScoreCard measures) predicted lower levels of obesity, poor diet, and tobacco use.
Kent et al, 2018 (12)	Developed tools to measure the culture of health and applied them to 32 organizations. The first tool was based on the Organizational Supports module of the ScoreCard and focused on the internal culture of health, programs, policies, and attributes of the physical and social environments that support employee health and well-being.	The internal culture of health survey based on the ScoreCard demonstrated adequate reliability and some validity in predicting outcome measures.
Linnan et al, 2019 (13)	Studied 2,843 US employers of various size and scope, selected from a Dun and Bradstreet database sample of 2.5 million private and public employers. All worksites employed at least 10 people.	Effectiveness of the measure was represented by the extent to which employers implemented ScoreCard interventions. Large employers were more likely to implement ScoreCard interventions in the workplace than small employers. Additionally, employers were increasing their commitment to ScoreCard and other intervention tools to promote health in the workplace.
Linnan et al, 2019 (44)	Conducted a national survey of occupational safety and health and workplace health promotion practitioners from 56 state and territorial health departments with 40 respondents; followed by in-depth interviews with a subset of survey respondents.	This study showed that the ScoreCard is widely accepted as a useful surveillance and implementation support tool. However, these activities have been limited by the significant resource constraints of occupational safety and health practitioners.
Macy et al, 2017 (43)	Administered the ScoreCard to a random sample of 1,200 worksites in Kentucky to collect cross-sectional data on employer health promotion practices. Study focused on depression inventions.	Too few Kentucky workplaces provide adequate health promotion interventions focused on depression management.
Meador et al, 2016 (13)	Compared 2 organization-level assessment and benchmarking tools, the ScoreCard and Prevention Partners’ WorkHealthy America. (https://data-anyware.com/PreventionPartners; no longer active). Examined data collected from 2013 to 2015 from both instruments to describe workplace health promotion practices across the US.	Study showed that these tools reached employers (N = 1,797) of all types and that many employers are using a comprehensive approach (85% of those using WorkHealthy America and 45% of those using the ScoreCard), increasing program effectiveness and impact
Onufrak et al, 2013 (51)	Collected data by using the summer wave of Porter Novelli's 2013 ConsumerStyles survey (49), which gathers information about health attitudes and behaviors. Interventions studied focused on nutrition and healthy eating in the workplace, such as the availability of healthy items in food/drink vending machines, cafeterias, or snack bars (if available at the worksite) and other healthy food environment support to encourage healthy eating habits among employees.	Survey items on workplace nutrition, physical activity habits, and overall wellness were based on the ScoreCard. Comprehensive workplace health promotions are rare, especially among small employers. Research is needed showing that improvements in ScoreCard values are correlated with improved eating habits, physical activity, and overall worker well-being.
Payne et al, 2018 (47)	Examined results from 41 employers that completed the ScoreCard in 2013 and 2015 and an employee survey as part of the National Healthy Worksite Program (46). Investigated the impact of a workplace culture of health elements on employee perceptions of organizational support for health and lifestyle risk.	Over the study timeframe, the organizations increased the number of interventions by an average of 27%. The organizations reported a doubling of their organizational commitment to and support of healthy worksite practices and a nearly doubling of support for programs advocating for employee well-being. The increase in support for healthy workplace practices was associated with an increase in perceptions of a positive culture of health among employees.
Safeer R et al, 2018 (48)	Twelve Johns Hopkins Medicine entities in Maryland, Washington, DC, and St. Petersburg, Florida, were evaluated by using the ScoreCard. Johns Hopkins Medicine used a dashboard system to track business unit scores on the various topic areas of the ScoreCard across entities and time and to identify opportunities for improvement.	The ScoreCard was shown to effectively measure and spur workplace health promotion improvements across this large organization. Eleven of 12 Johns Hopkins Medicine entities improved their overall score on the ScoreCard. The ScoreCard was also shown to be useful for helping large organizations with entities dispersed across various geographic locations implement a health promotion program uniformly while, at the same time, providing autonomy to each entity in addressing its unique needs and workplace culture.
Watkins et al, 2016 (42)	ScoreCard was administered to a random sample of 1,200 worksites in Kentucky, and cross-sectional data on employers’ general health promotion practices were collected.	Too few workplace programs in Kentucky are considered comprehensive.

Abbreviation: CDC, Centers for Disease Control and Prevention.

### Grey literature

Our grey literature search included publications of organizations outside the traditional academic or commercial publishing fields and distribution channels (eg, reports, government documents, white papers, case studies, evaluations). In addition to a general Google search (using the same search terms as the scientific literature search), we searched for reports from the CDC Workplace Health Resource Center (https://nccd.cdc.gov/WHRC/), state and local health departments, the database of Koop Award winners (www.thehealthproject.com), the Health Enhancement Research Organization (https://hero-health.org/), the National Institute for Occupational Safety and Health (https://www.cdc.gov/niosh/index.htm), the American Psychiatric Association Foundation (https://apafdn.org/), and other academic or community institutes engaged in workplace health. This search yielded 105 case study reports, of which 28 met our inclusion criteria (14–41). Each case study provided an overview of its workplace program, details of successes, challenges encountered, and insights on ScoreCard use and results.

### CDC ScoreCard database

CDC gave our study team access to its online survey database (launched in 2013), and we searched submissions from 2013 through 2020. The database review included examining the distribution of ScoreCard administrations across employer size groups, industry sectors, and states and the proportion of resubmissions and the change in average scores over time.

## Results

### Employer user characteristics

From 2013 through 2020, CDC received 4,681 online submissions of the ScoreCard from 2,484 unique employers in 48 states and the District of Columbia, Puerto Rico, and the US Virgin Islands. Large variations in the number of submissions per state (range, 0–744) suggested that some state or local health departments, or regional business groups, actively promoted the use of the ScoreCard, and some did not. Very small employers (1–100 employees) had the most submissions (54% of total), whereas large ones (≥751 employees) had the fewest (15.3%). Industries represented were the public sector (25.6%), private for-profit companies (53.6%), and private nonprofit organizations (20.8%). Submissions represented all 20 employer sectors of the North American Industry Classification System (www.census.gov/programs-surveys/economic-census/guidance/understanding-naics.html) (Figure), with the highest participation in the Health Care and Social Assistance (26.6%) and Educational Services (14.4%) categories. The greater uptake of the ScoreCard in Health Care and Social Assistance organizations was likely partly attributable to exposure to CDC in public health programs and to the ScoreCard in particular.

**Figure:**
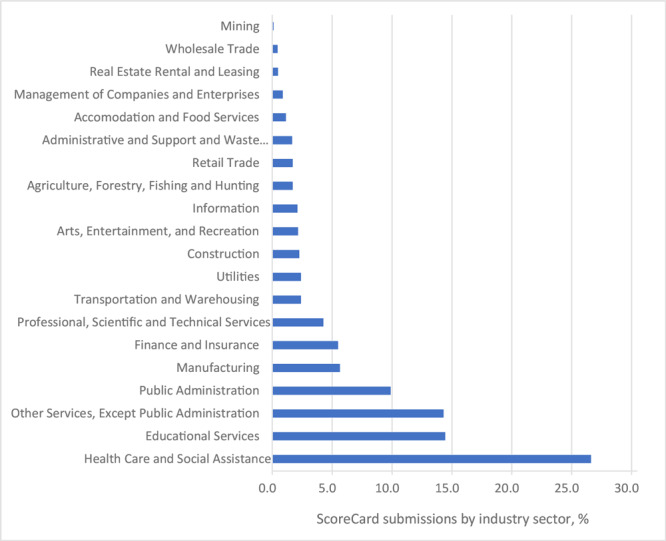
Distribution of CDC ScoreCard submissions, by employer Industry. Based on the North American Industry Classification System (www.census.gov/programs-surveys/economic-census/guidance/understanding-naics.html).

**Table:** 

Industry sector	Distribution of ScoreCard Submissions, %
Mining	0.1
Wholesale trade	0.4
Real estate rental and leasing	0.5
Management of companies and enterprises	0.9
Accommodation and food services	1.1
Administrative and support and waste management and remediation services	1.7
Retail trade	1.7
Agriculture, forestry, fishing, and hunting	1.7
Information	2.1
Arts, entertainment, and recreation	2.1
Construction	2.3
Utilities	2.4
Transportation and warehousing	2.4
Professional, scientific, and technical services	4.3
Finance and insurance	5.5
Manufacturing	5.6
Public administration	9.9
Other services, except public administration	14.3
Educational services	14.4
Health care and social assistance	26.6

In the completed online surveys submitted to CDC during the study period, employers most frequently reported having interventions in place related to insurance benefits (eg, free influenza vaccinations), interventions that are easy to implement (eg, food preparation, food storage facilities), and those subject to government regulations (eg, injury reporting systems). Interventions least likely to be in place were nutrition-related environmental supports and policies (eg, discounts on healthy food sold on-site). Although some of this deficit in program implementation may be attributable to lack of relevance (eg, food discounts were irrelevant to employers without cafeterias, vending machines, or catered food offerings), many employers possibly could not require their cafeteria or vending machine providers to offer healthy foods and snacks.

### Ways the CDC ScoreCard has been used

In examining the 12 peer-reviewed articles and 28 case studies that met our inclusion criteria, 4 general categories emerged that showed how the ScoreCard has been used in the public health, business, and research communities. These are 1) surveillance of employer workplace health practices, 2) training and technical assistance programs, 3) research studies on program effectiveness, and 4) employer efforts in monitoring, implementing, and evaluating their workplace health programs. These are not necessarily mutually exclusive categories, so a given article or case study may fit into more than one category.

**Surveillance efforts using the ScoreCard**. Two peer-reviewed articles reported on surveillance studies conducted by the state of Kentucky to examine employers’ health promotion practices. By using the ScoreCard, Watkins and colleagues determined that few worksites in Kentucky had workplace health programs, and even fewer had comprehensive programs (42). Small companies were less likely than large ones to have workplace health programs and less likely to start a program.

In a related study, Macy and colleagues (43) used data from the statewide assessment described by Watkins and colleagues (42) to examine the number of Kentucky workplaces currently offering screening, education, and treatment related to depression. The study also aimed to compare the number of Kentucky workplaces offering these program elements by size and industry type. Results showed that most worksites did not provide employee depression screening or education, counseling, or management training on identifying warning signs of depression or comprehensive treatment and follow-up services for depression. Small worksites (<250 employees) were less likely than larger ones to provide screening, education, counseling, training, and insurance coverage for depression. Overall, this study revealed a substantial gap in workplace health efforts targeted at depression.

Two other articles (13,44) used the ScoreCard to examine workplace health practices at the national level to measure the degree to which employers implemented best practices. Meador and colleagues examined data collected from 2013 through 2015 from the ScoreCard and another instrument (13). They found that 45% of ScoreCard users employed a comprehensive approach in their program initiatives. In studies conducted by Linnan and colleagues, larger worksites (≥500 employees) were more likely to implement workplace health initiatives than smaller worksites (<100 employees) (44). They also found through interviews with public health practitioners that the ScoreCard was widely viewed as a useful tool for surveillance and implementation support (45).

**Health promotion training and technical assistance**. Four peer-reviewed articles reported on CDC-led workplace health training and technical support programs that used the ScoreCard (8,9,46,47). In our grey literature review of 28 case studies of organizations, many of which participated in CDC support and outreach programs, the ScoreCard was used as a baseline assessment and planning tool and as a monitoring, goal-setting, and evaluation tool. Viewed in aggregate, these studies highlight the effectiveness of the ScoreCard in helping small and medium-sized companies build their workplace health promotion programs, particularly when used in conjunction with a training and technical assistance program (9). For example, employers that participated in the National Healthy Workplace Program, which offered free workplace health resources and technical assistance, improved their ScoreCard scores (46). However, we found little evidence that improved scores yielded reductions in the proportion of employees at high risk for poor nutrition, physical inactivity, high blood pressure, high cholesterol, high stress, or obesity. In many cases, the data were compromised by small noncohort samples and short study horizons.

Cluff and colleagues reported on CDC’s Work@Health Program, whose goals were to determine the best way to deliver employer training, increase employer knowledge of workplace health, and increase the number of evidence-based workplace health interventions at the worksite (8). The authors used a pretest and posttest design employing the ScoreCard and a survey of knowledge, attitudes, and behavior. In the intervention, employers received training in 1 of 3 formats (hands-on, online, or blended). The 8-module training curriculum guided participants through building an evidence-based workplace health program, followed by 6 to 10 months of technical assistance. The researchers found that mean knowledge scores after the training were higher than before training — a year after training, employers had significantly increased the number of evidence-based interventions in place (47.7 vs 35.5, *P* <. 001). Employer improvements did not significantly differ among the 3 training delivery formats.

**Research studies on program effectiveness**. Nearly two-thirds of the articles we reviewed highlighted the prominence of general organizational support initiatives that supported a culture of health in the workplace as an indicator of program effectiveness (10,11,13,43,47–49). For example, studies that correlated organizational support mechanisms and positive health outcomes showed that tangible and visible leadership support predicted lower levels of obesity, poor diet, tobacco use, and prescription drug use and improved self-reported physical activity, nutrition, and overall ScoreCard performance (10,11,47,48).

**Employer assessment, planning, and evaluation efforts**. Aside from the case studies associated with employer participation in CDC-led training and technical assistance programs, the study of Safeer and colleagues is an example of independent engagement by a large employer using the ScoreCard across its intended purposes (48). The authors concluded that the ScoreCard was an effective tool for providing overall accountability to measure and improve their workplace health programming.

## Discussion

Our examination of the current literature and available data on the ScoreCard — how it is used and its impact on workplace health — showed several gaps and opportunities.

**Surveillance of employer efforts.** Two studies showed the ScoreCard’s effectiveness in statewide surveillance of employer efforts (42,43). However, we could not determine how broadly these surveillance efforts were implemented. As in these studies, many broad applications may use separate platforms and may not be captured in the CDC-administered database. These studies also reinforced the likelihood that the ScoreCard database contains a self-selected sample of employers with an expressed interest in workplace health. Future research should include random-sample surveillance techniques to better understand general employer practices. Such findings would help local public health officials develop strategies to better engage businesses with targeted workplace health training and technical assistance based on their needs.

Through incentives such as grants or other funding opportunities, states and localities may be encouraged to employ the ScoreCard (by using the CDC online survey platform) as a surveillance tool to track employer efforts to improve worker health and align those data with relevant business results, such as absenteeism and health care use and costs. The ScoreCard can also be an essential dashboard metric for communities and states seeking to attract and retain qualified working-age adults and employers to business-friendly, healthy communities. CDC could also consider the tool as a complement to its Behavioral Risk Factor Surveillance System (https://www.cdc.gov/brfss/index.html), which is used routinely to assess population health at an individual level, by using the ScoreCard to assess workplace health.

**Low-wage industries and health equity.** The ScoreCard has not been effective at recruiting employers in low-wage industries (eg, retail, accommodation and food service, construction, mining), where workers tend to have a disproportionate number of health risks. Health risks for workers in these industries have been exacerbated by COVID-19 because of socioeconomic factors, such as crowded living conditions, lack of paid sick leave or health insurance, and employment in jobs at high risk for income loss (4,50). The substantial effect of wages on health status raises the question of health equity. One of the major barriers to engaging employers in socioeconomically underserved communities is a lack of resources (time, personnel, funds). Because it is a free resource, complemented by the CDC's training and technical support programs, the ScoreCard can offer guidance to employers on addressing employee health in such areas as preventive health screenings, paid sick leave, and access to vital health care services.

**Keeping the ScoreCard current.** CDC developed the ScoreCard with the assumption that a 5-year cycle of updates would be adequate. However, the unprecedented pace of both problems and innovative solutions related to employee health, safety, and well-being arising from the COVID-19 pandemic suggests a more accelerated schedule for tool enhancement is required to draw from the latest research and expert opinion to identify new important and relevant topics. For example, working from home for months and an increase in permanent telework led to a rise in mental health–related issues, such as loneliness, social isolation, and anxiety, at a time when employees have limited access to workplace health services and resources. Also, the ScoreCard’s Vaccine-Preventable Disease section could be expanded to explicitly include education about COVID-19 vaccine benefits, safety measures, and access to vaccination. These and other workplace developments may warrant more immediate revision of the tool. At the same time, common and persistent health risks that became more prevalent during the pandemic continue to be drivers of poor health outcomes. Attention to the latest research on evidence-based strategies to improve workers’ health, safety, and well-being is needed.

**In-depth study of ScoreCard.** An in-depth study is needed of how employers have used the tool in their day-to-day operations and the outcomes observed following the adoption of best and promising practices. More research is needed to show a cause–effect connection between improvements in ScoreCard values and improvements in organizational health, worker health and safety, and business outcomes (eg, health care costs, disease incidence, accidents, productivity, employee engagement, company stock price). Although some studies have already been conducted in this area, the body of evidence is still in its infancy and would benefit from long-term and rigorous study designs.

### Limitations

Our study had limitations. A key objective was to better understand how worksites used the ScoreCard in workplace health design, implementation, and evaluation. However, because of the self-selection nature of the instrument, aside from assembling submitted employer characteristics, the results of our review are not necessarily generalizable. Additionally, given the availability of the paper–pencil version of the ScoreCard, we could not determine how many employers downloaded the tool, used it offline, and did not submit their responses to the online database. The profile of users who decided to use the paper–pencil version of the ScoreCard is likely to differ from that of employers who completed the tool online.

### Conclusion

Since its release in 2012, real-world evidence has shown that the ScoreCard has been applied as designed and intended and has demonstrated its effectiveness in guiding workplace health efforts to promote employee health and safety, prevent disease, and manage chronic illness. Looking ahead, the tool’s usefulness should be leveraged and amplified to ensure it remains relevant, cutting edge, and evidence-based to support the needs of employers, researchers, and practitioners.

Overall, there are 4 major growth opportunities for the ScoreCard: 1) conducting large, random-sampling, surveillance studies; 2) meeting the needs of a changing and evolving workplace, with an emphasis on addressing SDOH and equity issues; 3) updating the ScoreCard with the latest research and addressing emergencies and the challenges and reality of rapid change (eg, the COVID-19 pandemic); and 4) tying organizational workplace health practices to health and business outcomes. Addressing these opportunities to enhance the utility of the ScoreCard under “new normal” work conditions is vital to assisting employers in improving employee health, safety, and well-being.
